# Retinoic acid-induced CHD5 upregulation and neuronal differentiation of neuroblastoma

**DOI:** 10.1186/s12943-015-0425-y

**Published:** 2015-08-07

**Authors:** Mayumi Higashi, Venkatadri Kolla, Radhika Iyer, Koumudi Naraparaju, Tiangang Zhuang, Sriharsha Kolla, Garrett M. Brodeur

**Affiliations:** Division of Oncology, Children’s Hospital of Philadelphia, and the Department of Pediatrics, University of Pennsylvania School of Medicine, CTRB Rm. 3018, 3501 Civic Center Blvd, Philadelphia, PA 19104 – 4302 USA; Department of Pediatric Surgery, Kyoto Prefectural University of Medicine, Kyoto, 602 – 8566 Japan

**Keywords:** Neuroblastoma, CHD5, Differentiation, Retinoic acid, TrkA

## Abstract

**Background:**

Chromodomain-helicase DNA binding protein 5 (CHD5) is an important tumor suppressor gene deleted from 1p36.31 in neuroblastomas (NBs). High CHD5 expression is associated with a favorable prognosis, but deletion or low expression is frequent in high-risk tumors. We explored the role of CHD5 expression in the neuronal differentiation of NB cell lines.

**Methods:**

NB cell lines SH-SY5Y (SY5Y), NGP, SK-N-DZ, IMR5, LAN5, SK-N-FI, NB69 and SH-EP were treated with 1–10 μM 13-cis-retinoic acid (13cRA) for 3–12 days. qRT-PCR and Western blot analyses were performed to measure mRNA and protein expression levels, respectively. Morphological differences were examined by both phase contrast and immunofluorescence studies.

**Results:**

Treatment of SY5Y cells with 13cRA caused upregulation of CHD5 expression in a time- and dose-dependent manner (1, 5, or 10 μM for 7 or 12 days) and also induced neuronal differentiation. Furthermore, both NGP and SK-N-DZ cells showed CHD5 upregulation and neuronal differentiation after 13cRA treatment. In contrast, 13cRA treatment of IMR5, LAN5, or SK-N-FI induced neither CHD5 expression nor neuronal differentiation. NB69 cells showed two different morphologies (neuronal and substrate adherent) after 12 days treatment with 10 μM of 13cRA. CHD5 expression was high in the neuronal cells, but low/absent in the flat, substrate adherent cells. Finally, NGF treatment caused upregulation of CHD5 expression and neuronal differentiation in SY5Y cells transfected to express TrkA (SY5Y-TrkA) but not in TrkA-null parental SY5Y cells, and both changes were blocked by a pan-TRK inhibitor.

**Conclusions:**

Treatment with 13cRA induces neuronal differentiation only in NB cells that upregulate CHD5. In addition, NGF induced CHD5 upregulation and neuronal differentiation only in TrkA expressing cells. Together, these results suggest that CHD5 is downstream of TrkA, and CHD5 expression may be crucial for neuronal differentiation induced by either 13cRA or TrkA/NGF signaling.

**Electronic supplementary material:**

The online version of this article (doi:10.1186/s12943-015-0425-y) contains supplementary material, which is available to authorized users.

## Background

Deletion of 1p36 is one of the most common genetic changes in neuroblastomas (NBs), and 1p deletion is strongly associated with *MYCN* amplification, advanced stages of disease and a poor prognosis [1]. Deletion of 1p36 is also found in many other malignancies, including adult cancers, and its loss is also associated with a poor prognosis in these tumors [2–5]. One or more tumor suppressor genes (TSGs) presumably reside in this deleted region. We narrowed the smallest region of deletion (SRD) in over 1,200 NBs to a <2 Mb region of 1p36.31, and we identified the gene encoding chromodomain-helicase-DNA binding protein 5 (*CHD5*) as the most likely TSG deleted in this region [6–8].

CHD5 is the fifth member of the nine-member CHD family, and it is most homologous to CHD3 and CHD4, which form a NuRD-type chromatin-remodeling complex [9–11]. CHD3 and CHD4 are expressed ubiquitously, but high *CHD5* expression is restricted to the nervous system and testis, suggesting that CHD5 has a role in regulating the development of these tissues [7, 9, 12, 13]. There are reports that *CHD5* acts as a TSG in other cancers, such as breast, colon, lung prostate, ovary and others [10, 14–25]. Previously, we reported that high *CHD5* expression was correlated with favorable outcome in NBs, whereas expression was low or absent in high-risk NBs [6, 26]. We also reported that the promoter of *CHD5* is frequently methylated in NBs with low or absent *CHD5* expression [6, 26], and suppression of *CHD5* expression by promoter methylation has been found in other cancers as well [18, 21, 22, 27]. *CHD5* expression can suppress the growth of many types of cancers, which suggests it is an important TSG in many forms of neoplasia [10]. *CHD5* expression in the testis is responsible for the histone to protamine switch in spermatogenesis [12, 28, 29]. However, the function of *CHD5* in neuronal cells and other tissues is unknown.

NBs are derived from sympathoadrenal precursors, and several pathways have been implicated in the neuronal differentiation of these cells, including nerve growth factor (NGF) and its cognate receptor TrkA. TrkA-expressing sympathetic neurons from newborn animals differentiate in vitro in the presence of NGF but undergo apoptosis in its absence [30]. Similarly, TrkA-expressing NB cells differentiate and survive for months when grown in vitro with NGF, but they also undergo apoptosis and die within a week without NGF [31]. Retinoids have also been shown to induce neuronal differentiation in NB cells in culture [32–34]. However, it is unclear if retinoids act directly through retinoic acid receptors, or indirectly by regulating the expression of other genes and proteins.

In this study, we showed that treatment of NB cells with 13-cis retinoic acid (13cRA) caused increased *CHD5* expression, which was consistently associated with neuronal differentiation. 13cRA induces gene expression changes as well as morphological differentiation based on the cell type. Our analysis also showed a direct correlation between *CHD5* expression and neuronal differentiation involving TrkA signaling, induced by NGF treatment in NB cells. Our results strongly suggest that *CHD5* expression plays an important role in neuronal differentiation induced by both 13cRA and NGF/TrkA signaling.

## Results

### Neuronal differentiation and upregulation of CHD5 Expression by 13cRA

**Fig. 1:**
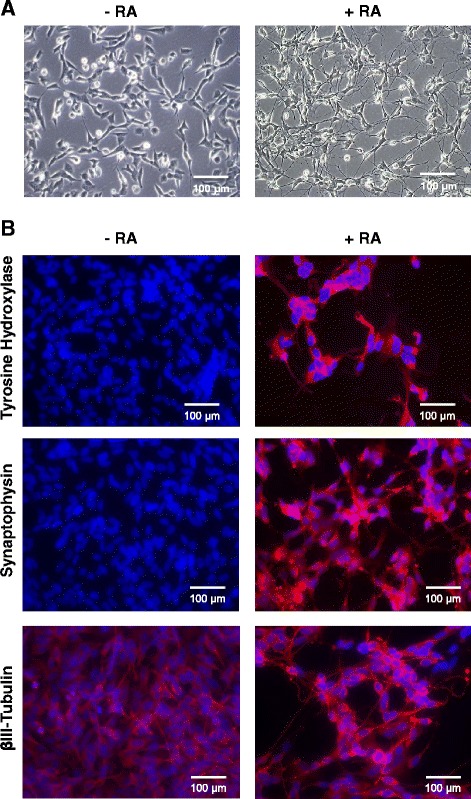
Neuronal differentiation of SY5Y cells with 13cRA treatment. (**a**) Representative phase contrast images showing SY5Y cell morphology change with or without 13cRA treatment (10 μM, 12 days). Multiple long neurites were observed in the presence of 13cRA. (**b**) Immunofluorescence of SY5Y cells with or without 13cRA treatment. Representative immunofluorescence images obtained when cells were treated with 13cRA and stained with neuronal markers tyrosine hydroxylase (TH), synaptophysin (SYP), and βIII-Tubulin. Control cells did not show significant staining with TH or SYP, but there was some βIII-Tubulin staining

To determine if there were changes in CHD5 expression in response to 13cRA treatment, we treated SY5Y cells (a neuronal subclone of SK-N-SH) with 1 μM, 5 μM or 10 μM 13cRA for 7 or 12 days and observed cells directly under the microscope. During the course of treatment, cells showed clear neurite extension in response to 13cRA (Fig. 1a). Representative phase contrast images were chosen to measure neurite outgrowth in the presence or absence of retinoic acid after 6 and 10 days (Additional file 1: Figure S1A). Neurite outgrowth was significantly longer in RA-treated SY5Y cultures compared to untreated controls (Additional file 1: Figure S1B). We also noticed a reduction in NB cell number upon retinoic acid treatment. These observations were confirmed by sulphorhodamine B (SRB) calorimetric assays in SY5Y, and also in the NB69 cell line (Additional file 2: Figure S2). To further validate neuronal differentiation in NB cells in response to 13cRA treatment, we performed immunofluorescence of SY5Y cells with neuronal markers: tyrosine hydroxylase (TH), synaptophysin (SYP) and βIII-Tubulin, in addition to the observations made through phase contrast microscopy. Cells treated with 13cRA showed clear increased expression for all the tested neuronal markers, whereas control cells showed limited or no expression (Fig. 1b). These results clearly show that 13cRA induced morphological and biochemical neuronal differentiation in SY5Y cells.

**Fig. 2:**
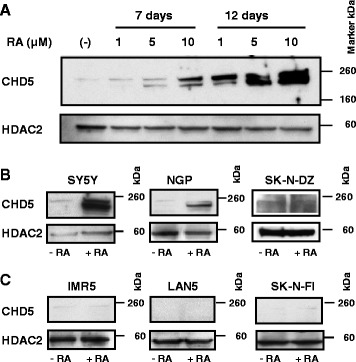
Upregulation of CHD5 expression in NB cells treated with 13cRA. (**a**) Representative immunoblot showing the upregulation of CHD5 from nuclear extracts of SY5Y cells treated with 13cRA at different concentrations and times. Western blot was stripped and probed with HDAC2 as an internal control for nuclear proteins, and we observed uniform HDAC2 expression. (**b**) and (**c**) Immunoblotting of CHD5. NB cell lines SY5Y, NGP, SK-N-DZ, IMR5, LAN5 and SK-N-FI were treated in the presence or absence of 10 μM 13cRA for 12 days and nuclear proteins were extracted. Western analysis showed CHD5 protein upregulation in SY5Y, NGP and SK-N-DZ cells (**b**), but not in IMR5, LAN5 and SK-N-FI (**c**). HDAC2 protein was used as an internal control for these blots

In addition, to assess the effects of 13cRA treatment on CHD5 expression, we performed western blot analyses. Immunoblotting of nuclear proteins for CHD5 showed dramatic upregulation of CHD5 by 13cRA in both a time- and dose- dependent manner. Blots were stripped and reprobed with HDAC2 as an internal control for nuclear protein (Fig. 2a). In order to confirm the association of increased CHD5 expression with neuronal differentiation, we extended our studies to additional NB cell lines. In addition to SY5Y, we treated NB cell lines NGP, SK-N-DZ, IMR5, LAN5, SK-N-FI, NB69 and SH-EP with 10 μM 13cRA for 12 days. Both SK-N-DZ and LAN5 cells showed cellular toxicity after a few days of 10 μM 13cRA treatment, so the dose was reduced to 5 μM 13cRA, and we treated both lines for only 3 days. We analyzed CHD5 protein expression in NB cells with or without 13cRA treatment by western blot analysis. Immunoblotting results are shown from three representative lines that responded to 13cRA exposure by undergoing neuronal differentiation (SY5Y, NGP, SK-N-DZ), and three that did not (IMR5, LAN5, SK-N-FI). Similar to SY5Y cells, NGP and SK-N-DZ cells showed clear upregulation of CHD5 protein by 13cRA treatment (Fig. 2b). However, IMR5, LAN5 and SK-N-FI cells showed no significant difference in CHD5 protein expression in response to 13cRA treatment (Fig. 2c), and no neuronal differentiation was observed (Table 1). We also treated SH-EP cells, a substrate-adherent (S-type) subclone of SK-N-SH, with 13cRA, but there was no appreciable change in either CHD5 expression or morphology (Table 1). These results suggest that increased CHD5 expression may be required for neuronal differentiation in response to 13cRA treatment.

**Table 1:** Summary of 1p and MYCN status, CHD5 mRNA expression, and morphology change in response to 13-cis retinoic acid in NB cell lines

Cell lines	1p status	MYCN status	CHD5 expression (WB or Q-PCR)		TrkA expression (Q-PCR)	Observed cell morphology change^a^
			Pre-RA	RA+	RA+	
SY5Y	No del	NA	+	↑	↑	Neuronal differentiation
NGP	Trans	A	-	↑	↑	Neuronal differentiation
SK-N-DZ	No del	A	-	↑	↑	Differentiation/death
IMR5	Del	A	-	→	→	No obvious change
LAN5	Del	A	-	→	→	Cell death
SK-N-FI	No del	NA	-	→	→	No obvious change
NB69	Del	NA	+	↓	ND	N- and S-type cells after RA treatment
SH-EP	No del	NA	-	→	ND	S-type. No obvious change

*Del* Deleted, *No del* Not deleted, *Trans* Translocated, *A* Amplified, *NA* Not amplified, *RA* Retinoic acid, *ND* Not done

^a^All cells were N-type and remained so except for SH-EP (S-type) and NB69 after RA treatment

### Neuronal differentiation of NB69 cells by 13cRA

Interestingly, the NB69 cell line showed a unique response to the 13cRA treatment. Unexposed NB69 cells exhibited a round, tightly clustered and semi-adherent phenotype (Additional file 3: Figure S3a). Treatment with 10 μM 13cRA induced a change in morphology after 1 day. However, after 7 days of treatment, there were two very different morphologic populations of cells—a smaller population of cells with a neuronal morphology and neurite outgrowth, and a larger population that were flat and substrate adherent (Additional file 3: Figure S3a). In order to further explore neuronal differentiation in NB cells upon 13cRA treatment, we performed immunofluorescence of NB69 cells with neuronal markers—TH, SYP and βIII-Tubulin—before and after 13cRA treatment. Cells treated with 13cRA showed neurite outgrowth and increased expression of neuronal markers exclusively in the neuronal cells (Additional file 3: Figure S3b). Additional morphological and biochemical evidence of neuronal differentiation in the neuronal subtype are also shown (Additional file 2: Figure S2 and Additional file 4: Figure S4). These results further suggest an association between CHD5 upregulation and neuronal differentiation in response to 13cRA in NB cells.

### Relationship between CHD5 and TrkA expression

**Fig. 3:**
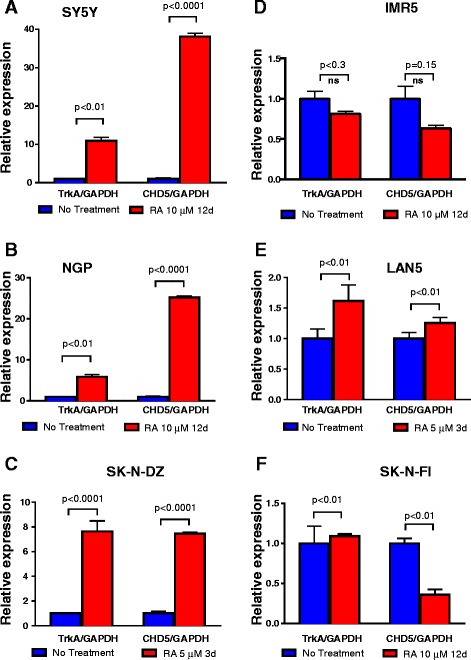
TrkA and CHD5 expression in NB cell lines. NB cell lines were treated with 5 or 10 μM of 13cRA for 3, or 12 days as indicated. CHD5 and TrkA was upregulated in (**a**) SY5Y, (**b**) NGP and (**c**) SK-N-DZ cell lines whereas (**d**) IMR5, (**e**) LAN5, and (**f**) SK-N-FI cells did not show a significant change in either CHD5 or TrkA gene expression upon with 13cRA treatment. Values were normalized (GAPDH) and calculated as relative levels of CHD5 and TrkA expression in untreated samples. The graphs show the results of at least three independent experiments in triplicates (SD). Statistical analysis was performed using one-way ANOVA using Prism followed by Tukey’s post-test and P-values were reported as shown. ns = not significant

To further investigate the relationship between CHD5 expression and neuronal differentiation, we analyzed the expression patterns of CHD5 and TrkA in our panel of NB lines (SY5Y, NGP, SK-N-DZ, IMR5, LAN5, and SK-N-FI) with or without 13cRA treatment (Fig. 3). Upregulation of both CHD5 and TrkA were found in the same cell lines that showed neuronal differentiation. SY5Y cells showed a significant increase over baseline expression for both TrkA and CHD5 in response to 13cRA (Fig. 3a). Similar, significant increases in TrkA and CHD5 expression were observed in NGP and SK-N-DZ in response to 13cRA with NGP (Fig. 3b, c). The other cell lines—IMR5, LAN5, and SK-N-FI—did not show significant changes in either CHD5 or TrkA gene expression with 13cRA treatment and did not differentiate (Fig. 3d, e, f). These results suggest that upregulation of both CHD5 and TrkA expression was required for cells to undergo morphological differentiation in response to 13cRA treatment.

### Increased CHD5 Expression by NGF-induced Activation of TrkA

**Fig. 4:**
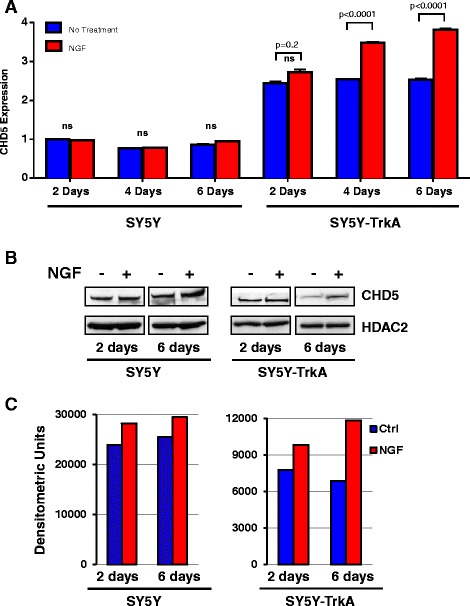
Effect of NGF on CHD5 expression. (**a**) CHD5 expression in SY5Y and SY5Y-TrkA cells with or without NGF (50 ng/ml) treatment. The values for CHD5 expression were calculated relative to the level of CHD5 expression in SY5Y on day 2 without NGF treatment. The graph shows the results of three independent experiments (SD). Statistical analysis was performed using two-way ANOVA using Prism followed by a Sidak post-test and P values were reported as shown. ns = not significant. (**b**) Protein expression of CHD5 and HDAC2. (**c**) Densitometric analysis of CHD5 protein expression in SY5Y-TrkA cells. The number of pixels from each band obtained from a representative Western blot were measured, and a bar graph was created using Prism to indicate the difference in CHD5 expression in the control and NGF treated cultures

To analyze the association between the NGF-TrkA signaling pathway and CHD5 expression, we treated SY5Y and SY5Y-TrkA cells with NGF, the cognate ligand of TrkA. Cells were harvested after 2, 4 and 6 days of NGF treatment, and CHD5 expression was analyzed by qPCR and western blot. Before NGF treatment, SY5Y-TrkA cells showed about 2.5 fold higher expression of CHD5 compared to the parental SY5Y cell line. Treatment with NGF (50 ng/ml) induced neuronal differentiation and significantly increased the CHD5 expression in a time-dependent manner in SY5Y-TrkA cells, whereas the parental SY5Y cells did not show any change in morphology or CHD5 expression in response to NGF (Fig. 4a). Similar results in were obtained in both lines with CHD5 protein expression upon retinoic acid treatment (Fig. 4b). Densitometric analysis of representative CHD5 protein expression data are presented as supplementary data (Fig. 4c).

**Fig. 5:**
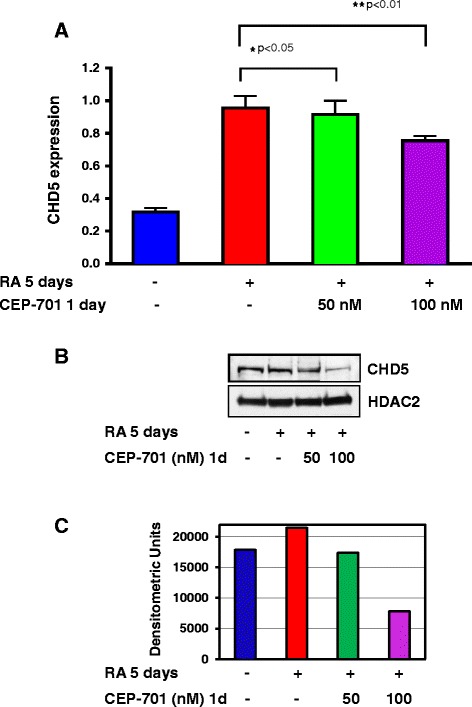
Effect of the TRK inhibitor CEP-701 on RA induced CHD5 expression. (**a**) CHD5 expression in SY-5Y-TrkA cells with or without RA and in the presence or absence of CEP-701. The values for CEP-701 inhibition were calculated relative to the levels of CHD5 expression upon 13cRA treatment. The graph shows the results of three independent experiments. Statistical analysis was performed using one-way ANOVA using Prism followed by Tukey’s post-test and P-values were reported as shown. (**b**) Protein expression of CHD5 and HDAC2. (**c**) Densitometric analysis of CHD5 protein expression in SY5Y-TrkA cells. The number of pixels from each band obtained from a representative Western blot were measured, and a bar graph was created using Prism to indicate the difference in CHD5 expression between control, 13cRA and CEP-701 treatments

To confirm that CHD5 upregulation by 13cRA was a result of the activation of TrkA, we used CEP701 (lestaurtinib), a potent TrkA inhibitor, to block the upregulation of TrkA expression by 13cRA in SY5Y-TrkA cells (Fig. 5). Cells were treated with 13cRA for 4 days, and then 50 nM or 100 nM of CEP701 were added to the medium with 13cRA for an additional day. Our results indicate that the CHD5 expression was upregulated after 5 days of 13cRA treatment, but the expression was suppressed in a dose-dependent manner by CEP701 treatment (Fig. 5a), with a corresponding inhibition of neuronal differentiation, as demonstrated by a decrease in neurite extension (Additional file 5: Figure S5). In addition to inhibition of neuronal differentiation we also noticed some growth inhibition or cell death at 100 nM CEP-701 concentrations that is known to block TrkA signaling and induce cell death. Similar results were obtained with CHD5 protein expression upon retinoic acid treatment (Fig. 5b). Densitometric analysis of representative CHD5 protein expression data was presented to indicate the difference in CHD5 expression between control, 13cRA and CEP-701 treatments (Fig. 5c). These results suggest that CHD5 expression is regulated downstream of NGF-TrkA signaling cascade.

## Discussion

In this study, we observed neuronal differentiation of NB cell lines in response to 13cRA, which was associated with upregulation of TrkA and CHD5 (Table 1). 13cRA is well studied as a model for inducing neuronal differentiation in some NB cell lines, and it has also been used in clinical treatment of high-risk NB [33, 35, 36]. However, not all NB cells show neuronal differentiation in response to 13cRA, and the mechanisms regulating 13cRA responsiveness are not understood. Ross and colleagues [37] classified NB cell morphology into three types; N-type for neuroblastic precursors, S-type for non-neuronal, substrate-adherent Schwannian precursors, and I-type for intermediate between N- and S-type. They also stated that N-type and I-type cells show a differentiation response to the 13cRA treatment, but S-type cells do not [38]. Our studies show that CHD5 expression is found in N-type cells (and not S-type cells, as seen in the NB69 or SH-EP line), and CHD5 expression is enhanced markedly by 13cRA treatment in N-type cells, concurrent with the neuronal differentiation. However, some cell lines, especially those with 1p deletion, do not undergo neuronal differentiation in response to 13cRA, and CHD5 expression is usually very low or absent in these cells.

Retinoids like 13cRA exert their function through the retinoic acid receptors, RAR and RXR. In the presence of 13cRA, RXR and RAR form dimers and bind to a DNA retinoic acid response element (RARE) [33, 35, 39]. The typical RARE sequence consists of two PuG(G/T)TCA motifs separated by a several nucleotides [39]. CHD5 has several partial RARE elements in the promoter sequence, but they are not the complete. Balmer and colleagues [40] classified RA-responsive genes into direct or indirect response groups. They defined direct regulation by RA as gene response (increase in expression) within 6 h of treatment with 1 μM RA, and the directly regulated genes had a typical RARE in its promoter. CHD5 does not fit in these criteria, since the response to 13cRA is longer than 1 day and it requires a higher dose of 13cRA for this response. This suggests that activation of CHD5 induced by 13cRA is an indirect response, although necessary for neuronal differentiation.

NB69 cells showed an interesting response to the 13cRA treatment. Cells showed two different types of morphology after 13cRA treatment, one is N-type and the other is S-type. CHD5 expression was high only in neuronal N-type cells, but very low in substrate-adherent S-type cells. Similar heterogeneity of cell morphology was found in the parental SK-N-SH NB cell line that had two different types of cells. The N-type was subcloned as the SH-SY5Y cell line, and S-type subcloned as the SH-EP cell line. Indeed, we saw neuronal differentiation in response to 13cRA only in SY5Y cells and not in SH-EP cells (Table 1), supporting the hypothesis that CHD5 expression is important for neuronal differentiation.

TrkA is also important for the neuronal differentiation of NB cells. In response to its cognate ligand NGF, TrkA induces neuronal differentiation in NBs [41, 42]. TrkA is also upregulated by 13cRA in some NB cell lines, but not in others, and this pattern correlated with the induction of CHD5 expression and with neuronal differentiation. We found marked TrkA upregulation following 13cRA treatment only in SY5Y, NGP and SK-N-DZ, the lines in which CHD5 expression was also markedly increased and that underwent neuronal differentiation. However, three other lines (IMR5, LAN5, SK-N-FI) showed neither CHD5 upregulation nor neuronal differentiation in response to 13cRA.

Previously, we had transfected CHD5 into several neuroblastoma lines (e.g., NLF, IMR5) [6], and although there was dramatic inhibition of growth, clonigenicity and tumorigenicity, we did not see any substantial morphological change or neuronal differentiation in these lines. Both IMR5 (shown here) and NLF (data not shown) are unresponsive to 13cRA, and the presence of increased levels of exogenous CHD5 alone did not produce neuronal differentiation in these lines. Taken together with our current results, this suggests that CHD5 expression may necessary but not sufficient to induce neuronal differentiation, as 13cRA likely has other effects in addition to upregulation of TrkA and CHD5 expression.

The role of CHD5 in neuronal differentiation of NB cells needs further investigation. Egan et al. reported a role of CHD5 in neurogenesis, and they showed that CHD5 directly interacts with trimethylated H3K27, regulating the expression of genes that are important for neuronal differentiation [13]. Considering the likely role of CHD5 as a component of a NuRD complex [10, 11, 43, 44] it could regulate neuronal differentiation by suppressing the expression of growth-related genes or inducing the expression of differentiation-related genes. Potts et al. have also shown the importance of CHD5 expression in neurons, and its depletion is linked to Alzheimer’s disease [43]. In addition, CHD5 expression is induced specifically in neuronal progenitors, indicating a role for neuronal differentiation and maturation, based on its expression patterns [45]. Thus, there is growing evidence that CHD5 has an important role in the regulation of neuronal differentiation, as well as in tumor suppression.

## Conclusions

Overall, our results suggest that CHD5 expression and TrkA expression are both downstream of the pathway of gene activation and neuronal differentiation induced by 13cRA. CHD5 gene expression is also upregulated by NGF in TrkA-transfected SY5Y cells, but not in TrkA-null parental SY5Y cells. Although it is not clear if the NGF-TrkA pathway is the main mechanism of CHD5 regulation, our data suggest that CHD5 is upregulated downstream of the NGF-TrkA pathway.

## Materials and methods

### Cell lines and treatment with 13cRA and NGF

NB cell lines SH-SY5Y (SY5Y), NGP, SK-N-DZ, IMR5, LAN5, SK-N-FI, NB69 and SH-EP were cultured in RPMI 1640 medium with 10 % FBS at 37 °C, and 5 % CO_2_. For the 13cRA treatment of cells, 13cRA (Sigma-Aldrich, St. Louis, MO) was added to the medium at different concentrations, and the medium was changed daily. Also, we previously established an SY5Y clone (SY5Y-TrkA) that constitutively expresses TrkA [46]. For NGF treatment of cells, hNGF (PeproTech, Rocky Hill, NJ) was added to the medium at the concentration of 50 ng/ml, and the medium was changed every two days. For the analysis of TrkA inhibition, CEP701 (lestaurtinib, Cephalon Inc. Frazer, PA) was added to the culture medium.

### Immunoblotting

Cells were harvested with 0.05 % trypsin after incubation in each condition. Protein was extracted using NE-PER® Nuclear and Cytoplasmic Extraction Reagents (Thermo–Fisher Scientific; Pittsburgh, PA). SDS-PAGE and transfer were performed with Invitrogen systems (Invitrogen–Life Technologies; Grand Island, NY). Immunoblotting was performed with the following primary and secondary antibodies and dilutions: anti-CHD5 antibody (Santa-Cruz, sc-68390; Santa Cruz Biotechnology, Inc.; Dallas, TX) 1:1000, anti-HDAC2 antibody (Santa-Cruz, sc-6296) 1:1000, ECL-donkey anti-rabbit IgG-HRP (GE NA934V; GE Healthcare Life Sciences; Pittsburgh, PA) 1:2500, donkey anti-goat IgG-HRP (Santa-Cruz, sc-2020) 1:5000. For each blot, the reaction was performed at 4 °C overnight for the primary antibody and at room temperature for 2 h for the secondary antibody.

### Real-time quantitative RT-PCR (Q-PCR)

The SYBR green system with SYBR® Green PCR Master Mix (Applied Biosystems; Grand Island, NY) was used for analysis of gene expression by qPCR. Primer sets for *CHD5* TrkA/*NTRK1* and *GAPDH* are shown in Additional file 6: Table S1. All samples were run triplicate, and each experiment was conducted at least 3 times. Values were calculated as relative rates from a standard curve, and GAPDH was used as an internal control. PCR was run on a 7900HT Fast Real-Time PCR System (Applied Biosystems, Grand Island, NY).

### Immunofluorescence

Cells were fixed on either 8-well chamber slides or 24-well culture plates with 4 % formaldehyde. Blocking was performed in PBS buffer with 0.1 % Triton X-100, 1 % BSA and 10 % FBS for 2 h. Primary and secondary antibody binding was performed in PBS with 0.5 % Triton X-100, 1 % BSA with the following concentrations: CHD5 (Santa-Cruz sc-68390) 1:500; Tyrosine Hydroxylase (Novocastra NCL-TH) 1:200; Synaptophysin (R&D Systems Inc.) 1:500; βIII-Tubulin (R&D Systems Inc.) 1:500; anti-mouse secondary antibody (Invitrogen Alexa Fluor 546) 1:500. Antibody binding was performed at 4 °C overnight with primary antibody and at room temperature for 2 h with secondary antibody. Prolong Gold Anti-fade reagent (Invitrogen, Palo Alto, CA) was used for DAPI staining to preserve fluorescence signal.

### Sulphorhodamine B (SRB) assay

Sulforhodamine B (SRB) assays were performed to determine the effect of 13cRA on NB cells. SY5Y and NB69 cells (5×10^3^/ well) in triplicates were plated in 96 well plates and treated with or without 10 μM 13cRA. Plates were harvested after 2, 5, and 7 days of 13cRA treatment and processed for SRB assays as per the standard protocol. Cell viability was analyzed by SRB assay by measuring optical density (OD) of bound dye. All experiments were performed in triplicate and repeated at least 3 times.

### Statistical analysis

Statistical analyses were performed using the Prism ANOVA method. One-way ANOVA was performed followed by Tukey’s post-test and two-way ANOVA followed by a Sidak post-test. Each experiment was performed at least three times and triplicate readings were used and reported all p-values. For all the CHD5 expression analysis with or without retinoic acid, NGF response and CEP-701 treatments values were compared between untreated and treated samples.

## Additional files

Additional file 1: Figure S1. (A) Neurite formation in SY5Y cells in response to 13cRA. Representative phase contrast images depicting extension of neurites in response to 13cRA treatment after 6 days and 10 days. Long neurites were observed when cells were treated with 13cRA. (B) Statistical analysis of neurite outgrowth following 13cRA treatment in SY5Y cells. Length of neurites from three to five representative fields of 13cRA treated cells were measured in μm and compared with neurites from untreated cells. Neurites were measured (μm-micrometer) from at least three independent experiments, and p-values were calculated by one-way ANOVA using Prism followed by Tukey’s post-test. (DOC 208 kb) Additional file 2: Figure S2. Effect of 13cRA on cell proliferation by SRB assay. SY5Y and NB69 cells were plated on 96 well plates and treated with or without 10 μM retinoic acid. Plates were harvested after 2, 5, and 7 days of 13cRA treatment. Cell viability was analyzed by an SRB assay measuring optical density (OD). Cell lines treated with 13cRA showed lower OD, indicating reduced cell number compared to untreated control cells. (DOC 46 kb) Additional file 3: Figure S3. NB69 morphology in response to 13cRA. (A) (A) Representative phase contrast images showing NB69 morphology change with or without 13cRA treatment (10 μM, 12 days). Two very different morphologic populations of cells were observed: a neuronal morphology with neurite outgrowth (N-type) (Red arrows), and flat and substrate adherent (S-type) (Blue arrows). (B) Immunofluorescence of NB69 cells with or without 13cRA treatment. Representative immunofluorescence images obtained when cells were treated with 13cRA and stained with neuronal markers TH, SYN, and βIII-Tubulin. (DOC 395 kb) Additional file 4: Figure S4 (A) Detection of neurites in NB69 cells in response to retinoic acid. Representative phase contrast images of NB69 cells showing long neurites upon 13cRA treatment for 6 and 10 days. (B) Statistical analysis of various neurites upon 13cRA treatment in NB69 cells. Length of neurites from various selected fields were measured in μm and compared with neurites from untreated cells. Neurites were measured (μm-micrometer) from at least three independent experiments and p-values were calculated by one-way ANOVA using Prism followed by Tukey’s post-test. (DOC 296 kb) Additional file 5: Figure S5. (A) Neurite inhibition by CEP-701 in SY5Y-TrkA cells. Representative phase contrast images of SY5Y-TrkA cells depicting extension of neurites in response to 13cRA treatment after 4 days, and subsequent neurite inhibition following 100 nM CEP-701 treatment (μm-micrometer) (B) Statistical analysis of neurite outgrowth following 13cRA as well as CEP-701 treatment of SY5Y-TrkA cells. Length of neurites (μm-micrometer) from three to five representative fields of 13cRA and CEP-701 treated cells were measured in μm and compared with neurites from untreated cells. P-values were calculated by one-way ANOVA using Prism followed by Tukey’s post-test. (DOC 148 kb) Additional file 6: Table S1. Nucleotide sequences of primers used in this study (DOCX 15 kb)

## Abbreviations

CHD5: Chromodomain-helicase-DNA binding protein 5
NGF: Nerve growth factor
NB: Neuroblastoma
13cRA: 13-*cis*-retinoic acid
