# Supplementary material for: Retinoic acid-induced CHD5 upregulation and neuronal differentiation of neuroblastoma
Source: Mol Cancer. 2015 Aug 7;14:150. doi: 10.1186/s12943-015-0425-y (PMC4527355; doi:10.1186/s12943-015-0425-y)
Supplement: Additional file 6: Table S1. — Nucleotide sequences of primers used in this study (DOCX 15 kb) [file 12943_2015_425_MOESM6_ESM.docx]

**Supplementary table S1.** Nucleotide sequences of primers used for qPCR amplification

|  | **Forward** | **Reverse** |
| --- | --- | --- |
| CHD5 | 5’CGAAGGCTACAAGTATGAGCGG3’ | 5’GGTTGAGAGGAGGAAGCAGAAC3’ |
| TrkA | 5’CACTAACAGCACATCTGGAGACC3’ | 5’TGAGCACAAGGAGCAGCGTAGA3’ |
| GAPDH | 5’GTCTCCTCTGACTTCAACAGCG3’ | 5’ACCACCCTGTTGCTGTAGCCAA3’ |
