# Supplementary material for: Retinoic acid-induced CHD5 upregulation and neuronal differentiation of neuroblastoma
Source: Mol Cancer. 2015 Aug 7;14:150. doi: 10.1186/s12943-015-0425-y (PMC4527355; doi:10.1186/s12943-015-0425-y)
Supplement: Supplementary file 2 [file 12943_2015_425_MOESM1_ESM.doc]

Figure S1

A

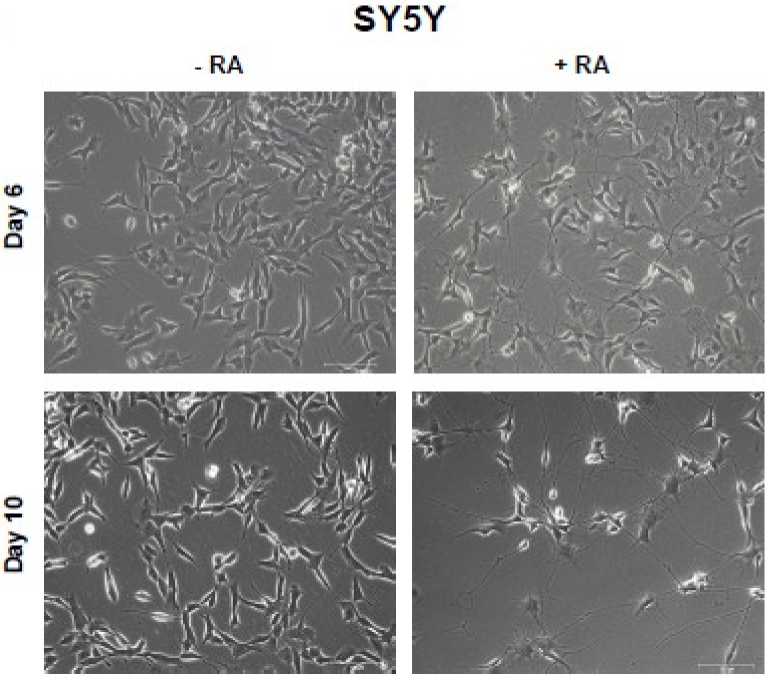
SY5Y
- RA
+ RA
Day 6
Day 10

B

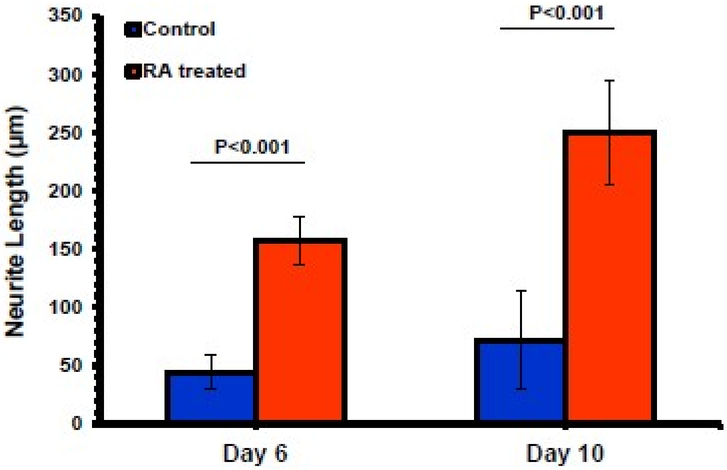
Control
RA treated
P<0.001
P<0.001
Neurite Length (μm)
350
300
250
200
150
100
50
0
Day 6
Day 10
