# Supplementary material for: Retinoic acid-induced CHD5 upregulation and neuronal differentiation of neuroblastoma
Source: Mol Cancer. 2015 Aug 7;14:150. doi: 10.1186/s12943-015-0425-y (PMC4527355; doi:10.1186/s12943-015-0425-y)
Supplement: Supplementary file 3 [file 12943_2015_425_MOESM2_ESM.doc]

Figure S2

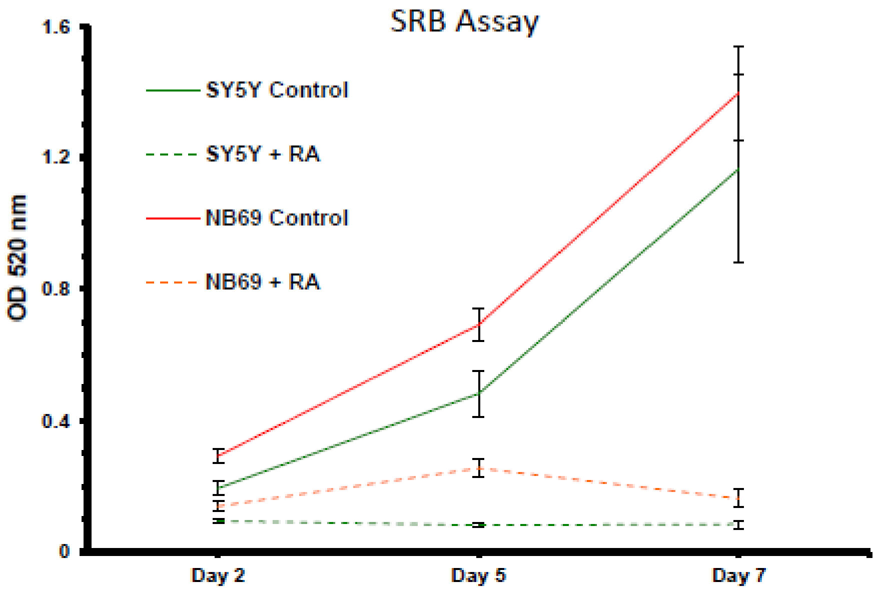
SRB Assay
OD 520 nm
1.6
1.2
0.8
0.4
0
SY5Y Control
SY5Y + RA
NB69 Control
NB69 + RA
Day 2
Day 5
Day 7
