# Supplementary material for: Retinoic acid-induced CHD5 upregulation and neuronal differentiation of neuroblastoma
Source: Mol Cancer. 2015 Aug 7;14:150. doi: 10.1186/s12943-015-0425-y (PMC4527355; doi:10.1186/s12943-015-0425-y)
Supplement: Supplementary file 4 [file 12943_2015_425_MOESM3_ESM.doc]

Figure S3

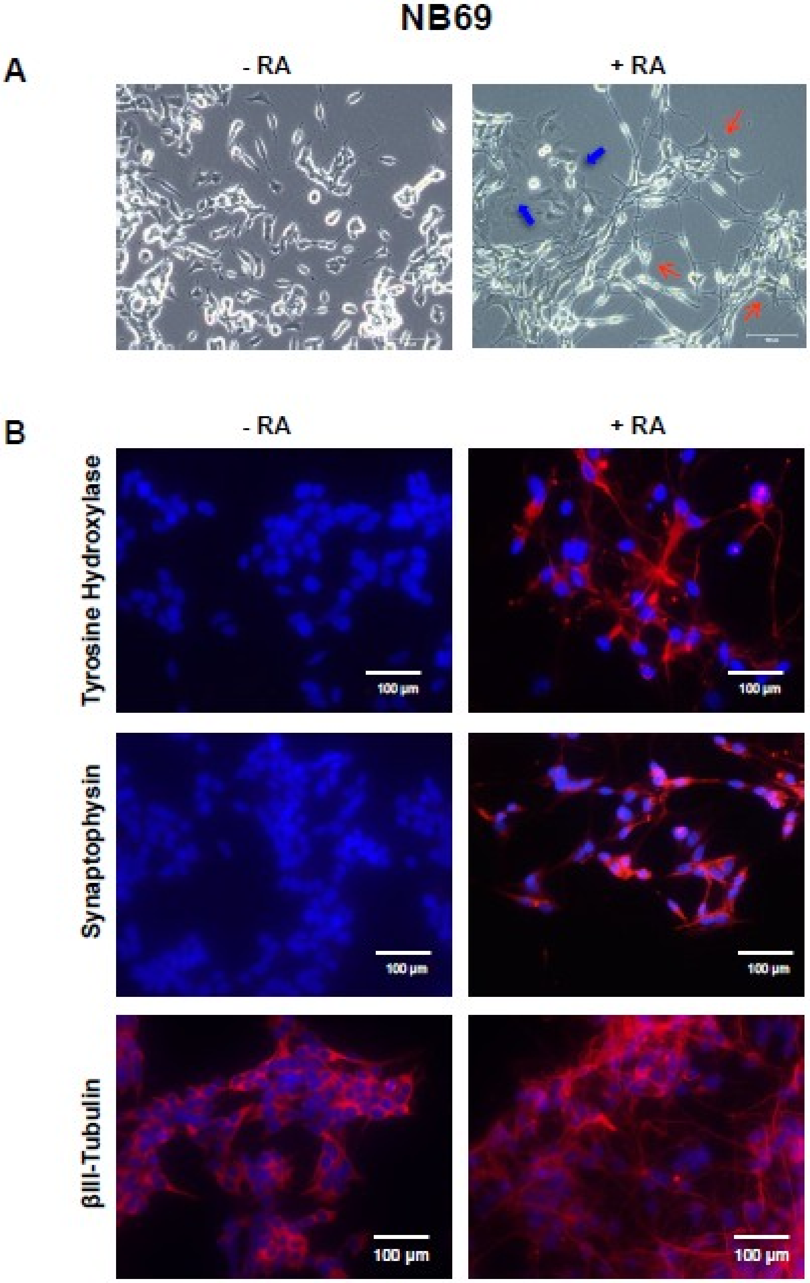
NB69
A
- RA
+ RA
B
- RA
+ RA
Tyrosine Hydroxylase
Synaptophysin
βIII-Tubulin
100 µm
100 µm
100 µm
100 µm
100 µm
100 µm
