# Supplementary material for: Retinoic acid-induced CHD5 upregulation and neuronal differentiation of neuroblastoma
Source: Mol Cancer. 2015 Aug 7;14:150. doi: 10.1186/s12943-015-0425-y (PMC4527355; doi:10.1186/s12943-015-0425-y)

Figure S4

NB69

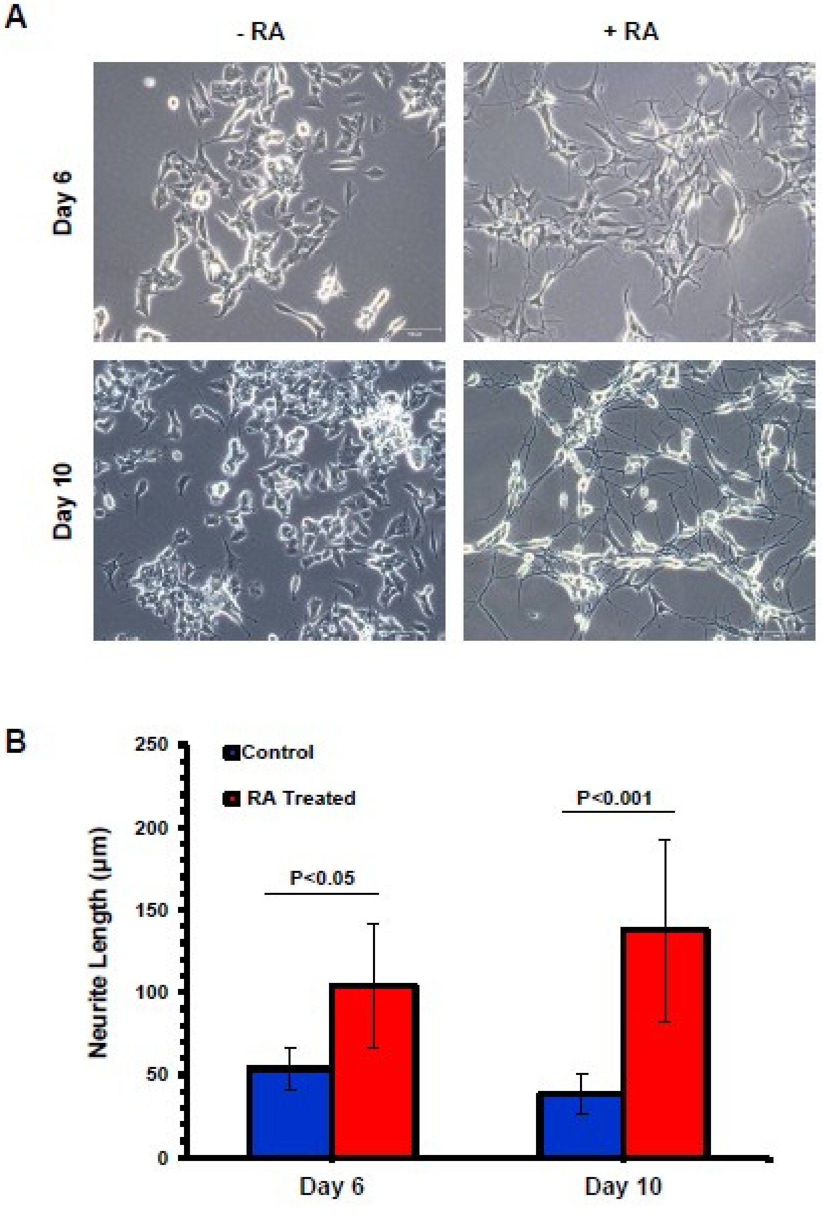

Supplement: Supplementary file 5 [file 12943_2015_425_MOESM4_ESM.doc]
